# Anti‐Pr antibody induced cold autoimmune hemolytic anemia following pneumococcal vaccination

**DOI:** 10.1002/ccr3.2365

**Published:** 2019-08-09

**Authors:** Laurel Bowen, Nichole LePage, Magdalena Lewandowska, Dan A. Waxman

**Affiliations:** ^1^ Department of Pathology and Laboratory Medicine Indiana University School of Medicine Indianapolis IN USA; ^2^ Department of Hematology Oncology Indiana University Health Indianapolis IN USA; ^3^ Versiti, Blood Center of Indiana Indianapolis IN USA

**Keywords:** anti‐Pr antibody, autoantibody, cold autoimmune hemolytic anemia, IgM, pneumococcal vaccination

## Abstract

While rare, vaccination‐induced autoantibodies can occur outside of the pediatric population. This diagnosis should be considered after infectious and lymphoproliferative disorders are ruled out. Clinical management will depend on the individual case, but all patients should be monitored closely.

## 1 INTRODUCTION

This is the first reported case of cold antibody autoimmune hemolytic anemia due to anti‐Pr autoantibodies attributed to Pneumococcal vaccination in an adult patient. The patient presented 1 week after receiving the vaccination with low hemoglobin, jaundice, and dyspnea with no prior history of anemia. Extensive workup including ruling out infectious and malignant causes was unremarkable, and his treatment consisted of transfusions and a course of steroids. An autoantibody anti‐Pr was identified with persistence but decreased reactivity up to 3 months after his initial presentation. Most cases of this identified autoantibody are associated with infectious causes or vaccination associated in the pediatric population.

Cold antibody autoimmune hemolytic anemia (AIHA) due to anti‐Pr autoantibodies is uncommon, with most cases being associated with infectious etiologies or lymphoproliferative disorders. A few cases have been attributed to vaccinations, and most of these have been reported in children. This is the first reported case of cold antibody AIHA in a 72‐year‐old man following pneumococcal vaccination with Prevnar 13.

## 2 CLINICAL PRESENTATION

The patient is a 72‐year‐old man who presented with jaundice and increasing dyspnea on exertion and was found to be anemic with hemoglobin of 7.4 g/dL. His symptoms began approximately 1 week after receiving the Prevnar 13 vaccination. The patient had been in stable health with a documented hemoglobin level of 13.9 g/dL at the time of his implantable cardioverter‐defibrillator (ICD) placement 2 months prior. His past medical history included congestive heart failure with ejection fraction of 10%, type II diabetes mellitus, and hypertension. His medication regimen had been stable for several years and included low strength aspirin, naproxen, lisinopril, carvedilol, furosemide, potassium chloride, simvastatin, metformin, glipizide, gabapentin, omeprazole, docusate sodium, folic acid, and a daily multivitamin. He had no prior history of anemia and had never before required a blood transfusion.

At presentation, the patient was jaundiced with stable vital signs (temperature of 98.8°F, heart rate of 88 beats/minute, blood pressure of 112/61 mm Hg, and oxygen saturation of 97% on room air). Laboratory results were significant for a low RBC count (2.60 M/CUMM; normal range: 4.15‐5.75 M/CUMM), reticulocyte count of 0.5 percent, elevated lactate dehydrogenase (LDH; 445 U/L; normal range 125‐243 U/L), decreased haptoglobin (<8 mg/dL; normal range 30‐200 mg/dL), and elevated total bilirubin (5.2 mg/dL; normal level up to 1.2 mg/dL). A peripheral blood smear was remarkable for spherocytosis. Direct antiglobulin testing was negative on two separate occasions for IgG as well as complement C3 components. A cold autoantibody was identified that reacted to all panel cells. The antibody was nonreactive with enzyme‐treated RBCs and identified as anti‐Pr The patient was diagnosed with cold antibody AIHA most likely secondary to recent administration of the pneumococcal vaccination Prevnar 13.

Further workup was unremarkable including the following: hepatitis serology testing, serum protein electrophoresis with immunofixation, general flow cytometry screening, Cryoglobulin testing, iron studies, folate, and B12. Parvovirus showed positive IgG and negative IgM, consistent with prior infection. Polymerase chain reaction testing for Parvovirus was negative. Computed tomography scan of the chest, abdomen, and pelvis demonstrated several borderline mediastinal lymph nodes measuring up to 1.3 cm, a left implantable cardioverter‐defibrillator (ICD) in place, a moderately enlarged heart, no obvious lung abnormalities or nodules, and a normal appearing liver and spleen with no abdominal or pelvic lymphadenopathy. He had a white blood cell count of 3.5 K/CUMM (normal range 3.3‐10.5 K/CUMM) and platelet count of 215 K/CUMM (normal range 150‐400 K/CUMM).

During his hospitalization, the patient's hemoglobin dropped to 6.6 g/dL, and his bilirubin increased to 6.6 mg/dL with a normal direct bilirubin of 0.5 mg/dL. The patient was transfused with one unit of packed red blood cells and discharged home in stable condition with a post‐transfusion hemoglobin level of 7.5 g/dL. At follow‐up, 11 days after his transfusion, his hemoglobin had dropped to 6.8 g/dL. He received two additional units of packed red blood cells with rise in hemoglobin to 9.3 g/dL. The patient was also treated with a course of prednisone with taper over 2 months. His hemoglobin level continued to rise, reaching 13.8 g/dL 3 months after discontinuation of steroid treatment. A blood sample at that time showed persistence of anti‐Pr, though with overall decreased reactivity strengths. The patient's laboratory studies showed no evidence of hemolysis with a normal LDH of 187 U/L and RBC of 4.61 M/CUMM.

## 3 CASE DISCUSSION

Cold autoantibody AIHA with Pr specificity is uncommon with the majority of cases occurring due to autoantibodies formed against the I/i blood groups. The antigenic determinants of the Pr antigen have been identified as O‐glycosidically linked disialo‐tetra/monosialo‐trisaccharides and are found on almost all RBCs at the distal N terminus of glycophorins.^1^ Hence, another characteristic of this group, helpful in antibody identification, is sensitivity to enzyme treatment. Most cases of auto–anti‐Pr red blood cell destruction have been reported in association with infections. These have included rubella, varicella, *Mycoplasma pneumoniae*, Epstein‐Barr virus, and cytomegalovirus.^2^, ^3^, ^4^ Cases preceded by infections are mostly transient and self‐limiting. Some cases become chronic with continued production of auto–anti‐Pr antibodies. Chronic cold hemagglutinin disease cases have been reported in association with lymphoproliferative disorders and in patients with no detectable underlying disease.^4^, ^5^

To our knowledge, this is the first reported case of anti‐Pr hemolytic anemia in the setting of recent pneumococcal vaccination with Prevnar 13. It is well established that vaccinations can elicit the formation of autoantibodies, including those with Pr specificity. In the 1980s, Colling and colleagues demonstrated cold agglutinin autoantibody production in rabbits immunized with Streptococcus pneumoniae type XIV, a serotype included in the Prevnar 13 vaccine.^6^ While not identified specifically as anti‐Pr, these antibodies were characterized as equally reactive to I‐ and i‐bearing human erythrocytes, a feature of this type. There are several case reports implicating vaccinations as the cause of AIHA in infants, children, and adolescents. A case of severe AIHA with IgM cold reactive autoantibodies with anti‐Pr specificity was reported in a 6‐week‐old infant 5 days after receiving the first dose of the diphtheria‐pertussis‐tetanus (DPT) vaccine.^7^ Other cases of AIHA have been described in association with the DPT vaccine,^8^, ^9^ the diphtheria‐tetanus‐typhoid vaccine,^10^ Typhoid fever vaccine, alone and in combination with the polio vaccine,^8^ oral polio vaccination,^11^ measles‐mumps‐rubella vaccination,^11^ as well as after receiving combinations of vaccinations for DPT, *Haemophilus influenza*, hepatitis B, and polio.^11^ Besides the case described above with anti‐Pr specificity, antibody specificity was not identified in these other cases. Fewer cases implicating vaccinations as the cause of AIHA have been reported in adults. There are four reports of adults, and one child, who developed AIHA following Influenza vaccination.^12^

Management of cold autoantibody AIHA is multimodal, and treatment decisions are often made on an individual basis. Our patient was initially stabilized with a single RBC transfusion during his acute presentation. He received an additional two units of RBCs and a course of prednisone during follow‐up as an outpatient. His hemoglobin gradually rose and returned to normal levels without any further treatments 5 months after initial presentation. Recent case reports of other patients with cold autoantibody AIHA with Pr specificity have been successfully treated with multimodal approaches that included plasmapheresis^13^ and combination immunotherapy with eculizumab, a terminal complement inhibitor, and rituximab.^14^

In summary, this report serves as a unique example of severe autoimmune hemolytic anemia from anti‐Pr that developed in the setting of pneumococcal vaccination with Prevnar 13. The patient suffered from severe AIHA requiring support with RBC transfusions. He also received a course of steroid therapy with prednisone, but it is unknown whether this directly impacted his clinical course. Clinicians should be aware of this potential complication in their patients following vaccinations.

## CONFLICT OF INTEREST

The authors declare no conflicts of interest.

## AUTHOR CONTRIBUTIONS

Laurel Bowen, MD: involved in drafting the manuscript with associated literature search and reviewing/revising submitted work. Nichole LePage, MD: involved in drafting and editing the manuscript, formatting, and reviewing/revising submitted work. Magdalena Lewandowska, MD: involved in providing the clinical presentation and associated laboratory values and reviewing/revising submitted work. Dan A. Waxman, MD: involved in providing the serological workup and interpretation and reviewing/revising submitted work.

## References

[1] Leo A , Kreft H , Hack H , Kempf T , Roelcke D . Restriction in the repertoire of the immunoglobulin light chain subgroup in pathological cold agglutinins with anti‐Pr specificity. Vox Sang. 2004;86:141‐147. 15023185 10.1111/j.0042-9007.2004.00401.x

[2] Schönitzer D , Kilga‐Nogler S , Trenkwalder B , LischH BC , Klein G , Hintner H . Autoimmune hemolysis caused by anti‐Pr (abstract). Infusionstherapie Und Klinische Ernahrung. 1985;12(4):181‐184. 4066004

[3] König A , Schabel A , Sugg U , Brand U , Roelcke D . Autoimmune hemolytic anemia caused by IgG λ‐monotypic cold agglutinins of anti‐Pr specificity after rubella infection. Transfusion. 2001;41:488‐492. 11316899 10.1046/j.1537-2995.2001.41040488.x

[4] Issitt PD , Anstee DJ . Applied Blood Group Serology (4th edn). Durham, NC: Montgomery Scientific Publications; 1998:853‐861.

[5] Silberstein LE , Robertson GA , Harris AC , Moreau L , Besa E , Nowell PC . Etiologic aspects of cold agglutinin disease: evidence for cytogenetically defined clones of lymphoid cells and the demonstration that an anti‐Pr cold autoantibody is derived from a chromosomally aberrant b cell clone. Blood. 1986;67:1705‐1709. 3486685

[6] Colling RG , Pearson TC , Brown JC . Association of bacterial carbohydrate‐specific cold agglutinin antibody production with immunization by group C, group B type III, and *Streptococcus Pneumoniae* type xiv streptococcal vaccines. Infect Immun. 1983;41:205‐213. 6345390 10.1128/iai.41.1.205-213.1983 PMC264764

[7] Johnson ST , McFarland JG , Kelly KJ , Casper JT , Gottschall JL . Transfusion support with RBCs from an Mk homozygote in a case of autoimmune hemolytic anemia following diphtheria‐pertussis‐tetanus vaccination. Transfusion. 2002;42:567‐571. 12084164 10.1046/j.1537-2995.2002.00093.x

[8] Zupanska B , Lawkowicz W , Gorska B , et al. Autoimmune hemolytic anemia in children. Br J Haematol. 1976;34:511‐520. 990186 10.1111/j.1365-2141.1976.tb03597.x

[9] Haneberg B , Matre R , Winsnes R , Dalen A , Vogt H , Finne PH . Acute hemolytic anemia related to diphtheria‐pertussis‐tetanus vaccination. Acta Paediatr Scand. 1978;67:345‐350. 654911 10.1111/j.1651-2227.1978.tb16332.x

[10] Gedikoglu G , Cantez T . Haemolytic‐anaemia relapses after immunization and pertussis. Lancet. 1967;2:894‐895. 10.1016/s0140-6736(67)92633-5 12389565

[11] Seltsam A , Shukry‐Schulz S , Salama A . Vaccination‐associated immune hemolytic anemia in two children. Transfusion. 2000;40:907‐909. 10960515 10.1046/j.1537-2995.2000.40080907.x

[12] Shizuma T . Autoimmune hemolytic anemia following Influenza virus infection or administration of Influenza vaccine. J Blood Disorders Transf. 2014;5:3.

[13] Cushing M , Degtyaryova D , Lomas‐Francis C . The role of plasmapheresis in the multimodal treatment of anti‐Pr cold agglutinin disease. Transfusion. 2010;50:2100‐2101. 21064265 10.1111/j.1537-2995.2010.02809.x

[14] Shapiro R , Chin‐Yee I , Lam S . Eculizumab as a bridge to immunosuppressive therapy in severe cold agglutinin disease of anti‐Pr specificity. Clinical Ca Rep. 2015;3(11):942‐944. 10.1002/ccr3.399 PMC4641479 26576277

